# Administration of Monoclonal Antibody for COVID-19 in Patient Homes

**DOI:** 10.1001/jamanetworkopen.2021.29388

**Published:** 2021-10-14

**Authors:** Anurag N. Malani, Beth LaVasseur, Jason Fair, Robert Domeier, Rita Vershum, Robin Fowler, Curtis D. Collins

**Affiliations:** 1 Department of Medicine, Section of Infectious Diseases, St Joseph Mercy Hospital, Ann Arbor, Michigan; 2 Department of Infection Prevention and Control, St Joseph Mercy Hospital, Ann Arbor, Michigan; 3 Department of Oncology, St Joseph Mercy Hospital, Ann Arbor, Michigan; 4 Huron Valley Ambulance, Ann Arbor, Michigan; 5 Department of Emergency Medicine, St Joseph Mercy Hospital, Ann Arbor, Michigan; 6 Department of Pharmacy, St Joseph Mercy Hospital, Ann Arbor, Michigan

## Abstract

This cohort study examines the outcomes of a program providing monoclonal antibody treatments to COVID-19–positive patients in their homes.

## Introduction

The COVID-19 pandemic has currently infected over 33 million people in the US, and over 2.2 million of those infected with the virus have been hospitalized.^1^ One important tool available for treatment of COVID-19 is neutralizing monoclonal antibodies (MAB). Bamlanivimab (LY-CoV555, Eli Lilly and Company), etesevimab (LY-CoV016, Eli Lilly and Company), and casirivimab/imdevimab (REGN-COV2, Regeneron Pharmaceuticals) were released through emergency use authorization (EUA) by the US Food and Drug Administration.^2,3^ MABs are recommended by both the National Institutes of Health and the Infectious Diseases Society of America for treatment of COVID-19 in ambulatory patients with mild or moderate COVID-19 with high risk for clinical progression.^2,3^

Following the EUA, several factors created implementation challenges for health systems. These included the administration location, infection control considerations, patient identification and enrollment processes, and workforce and resource considerations in facilities already responding to the winter surge of COVID-19. The approach for MAB administration has varied and is often facility specific. Strategies used have included administration in outpatient infusion centers and emergency departments (ED). During the recent spring surge of 2021, the Michigan Department of Human and Health Services reported that up to 30% of patients testing positive for COVID-19 may qualify for MAB and provided a goal that at least 50% of qualifying patients receive MAB.^4^ Descriptions of novel approaches to promote MAB administration are needed. One strategy implemented by our health system is partnership with community integrated paramedics (CIP) to promote home MAB administration. This cohort study describes our experience and how this strategy may factor into associated outcomes.

## Methods

From February to May 2021, a team of 3 nurses (B.L., R.V., and R.F.) reviewed new COVID-19–positive polymerase chain reaction tests and clinician referrals from multiple hospitals within the St Joseph Mercy Health System to assess EUA eligibility for MAB administration.^2,3^ The team contacted and obtained consent from eligible patients and worked with CIP to schedule home infusions. Nursing costs were $1250 weekly as they collectively worked approximately 25 hours per week at approximately $50 per hour. CIP obtained MAB and infusion supplies from the health system. The Michigan Department of Human and Health Services provided MAB for the health system at no cost. Standardized paramedicine protocols were developed for administration, and a medical director provided clinical support and expertise. The Centers for Medicare and Medicaid Services provided reimbursement for MAB administration for patients insured by Medicare at $310 per infusion, increasing to $750 per infusion in early May.^5^ The health system provided reimbursement to CIP for administration to patients without Medicare at the initial Medicare reimbursement rate, including patients without insurance. Patient demographic and clinical information was abstracted from electronic health records. Race and ethnicity and sex were defined by participant reporting in the health records. A 14-day follow-up period assessed outcomes following infusion.

This study was prepared in accordance with Strengthening the Reporting of Observational Studies in Epidemiology (STROBE) reporting guideline. This study was approved by the St Joseph Mercy Hospital institutional review board and determined to be exempt from patient consent with a waiver of HIPAA authorization. Data were analyzed using Microsoft Excel 365 version 2002 (Microsoft Corporation) and SPSS version 26 (IBM Corporation).

## Results

Among 144 patients administered MAB in the home (Figure), 83 (57.6%) were women and 125 (86.8%) were White individuals. The mean (SD) age was 60.1 (14.2) years (Table). Twenty patients (13.9%) were part of households where multiple members received infusions. Eight patients (5.6%) were hospitalized following home infusion because of worsening COVID-19 symptoms for a mean (SD) length of stay of 3.3 (2.2) days. None required intubation and all were successfully discharged. One patient (0.7%) developed a hypersensitivity reaction requiring hospitalization and 2 patients (1.4%) required an ED visit for hypersensitivity reactions without admission.

**Figure.**
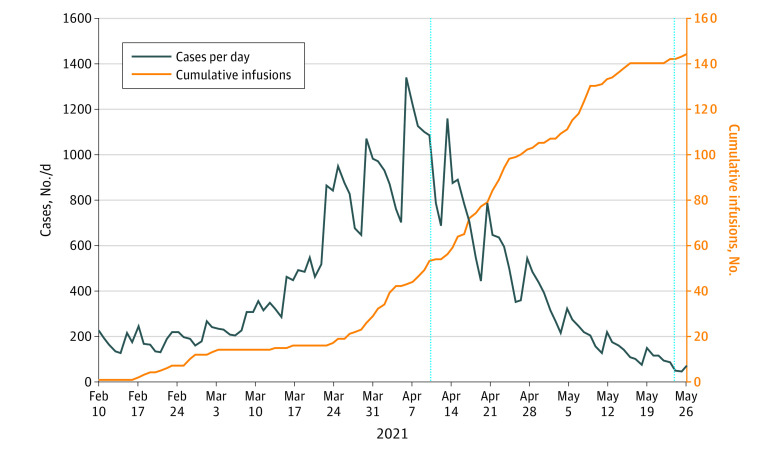
COVID-19 Cases by Day in Washtenaw, Wayne, and Livingston Counties, Michigan, and Cumulative Ambulatory Infusions Data taken from the state of Michigan database on COVID-19.^6^

**Table.** Demographics and Clinical Outcomes of Patients Administered Home Infusion of Monoclonal Antibodies

Variable	Patients, No. (%) (n = 144)
Sex	
Men	61 (42.4)
Women	83 (57.6)
Age ≥65 y	58 (40.3)
Mean (SD) age, y	60.1 (14.2)
Race	
Asian	1 (0.1)
Black	14 (9.7)
Hispanic or Latino	2 (1.4)
White	125 (86.8)
Unknown	2 (1.4)
Insurance	
Private	98 (68.1)
Medicare	34 (23.6)
Medicaid	7 (4.9)
Uninsured	5 (3.4)
Comorbid conditions by EUA indication^a^	
BMI ≥35	63 (43.8)
Median BMI (IQR)^b^	33 (27.5-40.3)
Chronic kidney disease	8 (5.6)
Diabetes	35 (24.3)
Immunosuppressive disease or receiving immunosuppressive therapy	12 (8.3)
With cardiovascular disease or hypertension and aged ≥55 y	63 (43.8)
With chronic obstructive pulmonary disease or other chronic respiratory disease and aged ≥55 y	22 (15.3)
COVID-19 symptoms	
Mild symptoms^c^	125 (86.8)
Moderate symptoms^d^	1 (0.7)
Both mild and moderate symptoms^c^^,^^d^	18 (12.5)
Time between symptoms and infusion, median (IQR), d	4 (3-6)
Monoclonal antibodies received	
Bamlanivimab and etesevimab	121 (84)
Bamlanivimab	19 (13.2)
Casivirimab with imdevimab	4 (2.8)
Hypersensitivity reactions	3 (2.1)
Adverse reactions requiring additional care	3 (2.1)
Hospitalized due to COVID-19 symptoms within 14 d and ≥12 h after infusion	8 (5.6)
Time between infusion and hospitalization, mean (SD), d^e^	2.1 (2.2)
No. of patients intubated^e^	0
Length of stay, mean (SD), d^e^	3.3 (2.2)
Death within 14 d of infusion	0

Abbreviations: BMI, body mass index (calculated as weight in kilograms divided by height in meters squared); EUA, emergency use authorization; IQR, interquartile range.

^a^ Patients may have had multiple comorbidities.

^b^ Analysis conducted on 137 patients with available data.

^c^ Mild symptoms include loss of taste or smell, fever, cough, sore throat, malaise, headache, muscle pain, nausea, vomiting, diarrhea, or abdominal pain.

^d^ Moderate symptoms include evidence of lower respiratory tract disease with respiratory symptoms or abnormal imaging.

^e^ Analysis performed on the subset of 8 patients requiring hospitalization.

## Discussion

During our recent COVID-19 surge in Michigan, home MAB infusion provided a critical framework to prevent high-risk patients from seeking ED care and requiring hospitalization. We provided MAB infusion to 144 patients, both insured and uninsured, in a successful partnership between the health system and CIP. Limitations included the inability to track the total number of eligible patients for MAB and outcomes for patients who refused treatment. At a time when physical and health care worker resources were significantly strained, our real-world approach leveraged the ability of nurses to identify, triage, and coordinate home MAB infusions while successfully preventing high risk patients from progression to severe disease and hospitalization.
